# Factors Associated with Attitude towards Wife-Beating among Married Women of the Reproductive Ages in Arba Minch Town, Southern Ethiopia: A Community-Based Cross-Sectional Study

**DOI:** 10.1155/2021/9980268

**Published:** 2021-09-13

**Authors:** Eshetu Andarge, Zeleke Gebru, Yordanos Sisay, Yohannes Shiferaw

**Affiliations:** ^1^School of Public Health, College of Medicine and Health Sciences, Arba Minch University, Arba Minch, P.O. Box: 021, Ethiopia; ^2^School of Public Health, College of Medicine and Health Sciences, Woliata Sodo University, Wolaita, P.O. Box: 168, Ethiopia; ^3^Department of Health Extension, Arba Minch College of Health Sciences, Arba Minch, P.O. Box: 155, Ethiopia

## Abstract

Evidence from demographic and health surveys in various countries and Ethiopia too showed that more women are generally believed to justify intimate partner violence (IPV) than men do. An attitude that justifies IPV is one of the factors affecting victimization and perpetration from IPV. However, women's justification about the violence and factors affecting the justification are not well documented, particularly by addressing household factors such as household food conditions. Therefore, the present study aims to fill this gap among married women of childbearing age so that evidence can be drawn for holistic interventions. A community-based cross-sectional study was conducted among 696 currently married women of childbearing age (15–49) by using a multistage cluster sampling technique to obtain the women from 11 kebeles (the smallest administrative unit in the government structure of Ethiopia) of Arba Minch town, Southern Ethiopia. Data were collected using a pretested and structured questionnaire. Logistic regression was performed using IBM SPSS version 20. The odds ratio with its 95% confidence interval was used to show the degree of association between the outcome variable and explanatory variables. Nearly two-thirds (59.5%) of the study women justified wife-beating in at least one of the five conditions. A higher odds of justification of wife-beating was observed among women whose marriage was arranged by any other person than the couples themselves, from food-insecure households, with a family size of 5 and above, in the age group of 30–39 years, and whose partner was in the age range of 31–39 years. In contrast, lower odds of justification of wife-beating was observed among women having an age difference of 10 or more years with their partner and those in a household wealth index of middle and higher category. Despite great efforts in realizing gender equality in the country, a higher proportion of women were having the attitude that justifies wife-beating in the five conditions specified to them. Interventions targeting the improvement of women's attitude towards wife-beating should target against the traditional norms of arranged marriage, improve household food conditions, and decrease family size.

## 1. Background

Intimate partner violence (IPV) is an act of violence against women which comes from an intimate partner and which takes the forms of violence including sexual, physical, and emotional abuse and a controlling behavior. It occurs predominantly among women from any background or irrespective of their socioeconomic, cultural, or religious backgrounds. On the other side, male mostly experience the violence act perpetuated by a stranger or an acquaintance than someone close to them [1]. Wife-beating is physical abuse of a woman by her present or former husband or male companion. This happens as a pattern of control of a wife by her husband, and it is often preceded by threats and verbal abuse making the wife to be solely dependent upon her husband owing to the legal, financial, and emotional ties that bind her to him. In some cases, they themselves believe that the beating has been caused by their actions and are filled with self-blame. Where they dare to report, they are trapped by an unresponsive legal system which does not effectively provide remedies to their claims against the men who seek to control them [2].

Even though wife-beating occurs everywhere in the world, the magnitude varies widely in countries within different income categories; a 4% versus 40% past 12 months' prevalence, respectively, was reported in many high-income countries and some low-income settings [3]. In Ethiopia, 68% of women believe that wife-beating can be justified in some occasions while many are unaware of the presence of laws against gender-based violence This might have resulted in few women seeking for support at times they face violence from their husbands [4]. Surprisingly, more women (30%) as compared to few men (5.6%) agreed on that there are justifiable reasons for a husband to hit his wife in at least four situations presented to them. .Unlike men, having children was associated with justifying wife-beating among women [5].

Wife-beating is also justified by most women in other sub-Saharan African countries. In Zimbabwe, 53% of women justified wife-beating in some conditions. In the same study, most women (more than 3 in 4) regarded wife-beating as a behavior that can be justified, and in more depth, in the focus group discussions, an occasional show of force was believed as a means to shape women who “misbehave” [6]. Consistent magnitudes of receptive attitudes were also reported from Uganda, Mali, and Ethiopia [3, 7].

Intimate partner violence could result in direct physical injury and indirect psychological impacts which would end up in various bad reproductive health outcomes and death [1]. Although intimate partner physical violence results in such shattering consequences, it is often underreported because of the influence of the deep-rooted cultural and social norms. These cultural and social norms, which are often supportive, create social standards for behaviors deemed as appropriate and inappropriate in the society. The social standards in turn govern behaviors as acceptable and unacceptable and co-ordinate interactions among the people living in that society. For instance, in some societies, women become vulnerable to violence by intimate partners because of the presence of societal traditional beliefs that provide men the right to control or discipline woman through physical means [8].

The underreporting of domestic violence stems not only from the culture and norms in the community outside women but also because of their own acceptance of the violence acts. Because of the persuasive norms in the society, women develop a tolerant attitude towards these unspoken violent acts. In some cases, beating seems to have a grade where only very severe abuses are given the tone and meaning while nonsevere violence is endemic and neglected as unremarkable in thoughts of women themselves. Physical violence is also underreported because of the looseness of the law against intimate partner violence as occasional imprisonments pose a fear of retaliation by the perpetrators against the victims [3].

Existing literature revealed different factors associated with women's attitude towards wife-beating. Female sex, husband having full autonomy in household decisions, exposure to physical or sexual IPV, being at younger age, from a rural area, from a household with lower wealth, below secondary-level education, and in a least paid occupation were associated with an increased likelihood of justifying wife abuse [9–11].

Women become more intolerant of violence and have more mental ability when food insecurity is less of an issue [12]. Gender attitudes supportive of male dominance were associated with hunger among girls. Similarly, for both sexes, being in a condition of hunger (higher level of food insecurity) is associated with a more masculine gender attitude [13]. Acceptance of wife-beating or tolerance to IPV has also been strongly and consistently affected by low levels of education and low household wealth [7, 14]. This emanates from the socioeconomic disadvantages women face and the cultural and religious restrictions imposed on them [14].

Although there was a significant variation in attitude towards refusing wife-beating among different regions in Ethiopia, higher age, educational level, and access to media were consistently associated with a high level of refusal of wife-beating. In contrast, residing in a rural area, being in a marital union, and having high number of living children were associated with a low level of refusal of wife-beating [15]. A disparity in resource ownership at the household or societal level is also associated with more abuse. In contrast, women who are employed faced a higher risk of abuse, particularly in communities with relatively higher acceptance of wife-beating [16]. Although the usual assumption is that men's attitude determined their use of force in relationships, as discussed earlier, in some societies, women themselves were found to be submissive to the abusive acts; thus, a further investigation to the factors that affect their attitude on acceptance of men's violence is warranted [17].

The study tried to investigate food condition at household (household-level food insecurity) as a single independent factor for women's attitude towards wife-beating, which, to the best of our knowledge, has not yet been considered in our country's setup. Given the perseverance of IPV across the globe and in the developing world, the findings of this study would be of help to policy making and programme planning in the local context as well by extension to Ethiopia where women are subject to the harms of the predominant patriarchal culture and submissive to justifying IPV.

## 2. Methods

### 2.1. Setting and Design of the Study

This study was conducted in Arba Minch town, Southern Ethiopia, located 505 km to the south of Addis Ababa (the capital city of Ethiopia), from March 10–20, 2017. Arba Minch is the main town of the currently structured Gamo zone and the then Gamo Gofa zone in the Southern Nations, Nationalities, and People's Regional (SNNPR) state [18]. The study was conducted as part of a thematic project on “Intimate partner violence and household food security among currently married women in Arba Minch town: magnitude, attitude, and the associated factors.” The first part of it on “disparities in intimate partner violence among married women from food-secure and -insecure households” has already been published [19]. The present study employed a community-based comparative cross-sectional study among currently married women of childbearing age (15–49 years).

### 2.2. Sample Size

The sample size was calculated using similar assumptions in the previously published project [19]. It was calculated by using Epi Info version 7.0 software with consideration of 50% proportion of women who justify against wife-beating in the five occasions/expected proportion of intimate partner violence in the last 12 months among food-secure currently married women (because of an absence of a similar study in a similar setting), 95% confidence level, 80% power, the ratio of food-secure to food-insecure households (*r* = 2), and odds ratio (OR) = 2. With an additional consideration of design effect of 2 for cluster sampling and a nonresponse rate of 10%, a total sample of 737 married women of childbearing age were included in the study, making the total sample size 737.

#### 2.2.1. Participant Selection and Sampling Procedure

All reproductive-age currently married women in Arba Minch town were taken as the source population. Randomly selected currently married women who were living with their husbands from the four kebeles in the current year were taken as the study population. Women with difficulty in communication either due to serious illness or any impairment as well as those who were not permanent residents of the town (lived in the town for less than 6 months) were excluded from the study.

The study women were selected in multiple stages. Initially, four kebeles were selected from a total of 11 kebeles in Arba Minch town (kebele: the smallest administrative unit in the government structure of Ethiopia) [18]. Secondly, the study women were selected randomly from a sampling frame of households with currently married women who were living with their husbands from the four kebeles in the current year. Women in the four levels summed up to 8321 with varying numbers in each kebele. Based on the number of women in each kebele, the total sample size initially estimated was proportionally allocated to each study kebele. Ultimately, random sampling was employed using SPSS to identify the required number from the frame of households in each kebeles. Nonresponse was declared after two visits if the target woman was not present at her home or if she refuses, for any reason, to respond to the questions. In a situation when a household had two or more eligible subjects, only one was selected by the lottery method to control the potential intrahousehold correlation.

### 2.3. Data Collection Techniques and Instruments

Eight female students who completed their high school studies and residents of Arba Minch town collected the data. The data collection was supervised by two health officers (graduates of bachelor's degrees in public health). An intensive 2-day training was given to the data collection team with a specific focus on the significances of the study, the research question under investigation or the objectives, methodology, contents of the instrument, interviewing skills, and the meaning of each question. The training particularly emphasized the importance of interviewing women in a private place and referral of victims of violence with serious risk (women who had sign and symptoms of depression and or anxiety, having a serious physical injury, and suicidal and homicidal ideations) using lecturing, group discussion, and role-plays among the data collection team, the principal investigator, and facilitators. After being given the list of women to be interviewed in each kebele, the data collectors were guided by health development army leaders so that they can easily access the houses of each sampled woman.

An interviewer-administered, pretested, and structured questionnaire was used. Attitude towards intimate partner violence was measured using 5 questions adapted from the Ethiopian Demographic and Health Survey (EDHS) 2016 questions [20]. Household food insecurity was measured by using the FAO's Food Insecurity Experience Scale [21] which was validated for use in different social contexts. Intimate partner violence was measured by a questionnaire partly adapted from the standard WHO multicountry study questionnaire for assessing women's health and domestic violence [22].

### 2.4. Data Entry and Analysis

EpiData software V3.1 was used to code, check, and enter data. IBM SPSS statistics version 20 was used to clean and analyze data exported from the EpiData software. Descriptive statistics involving frequencies, percentages, mean, and standard deviations were computed to describe findings. To compare the difference in attitude among food-secure and -insecure households, cross tabulation was made and a chi-square-test was used to check for significance.

Binary logistic regression was conducted in steps. To select potential candidate variables which affect women's attitude towards wife-beating, initially, cross tabulation was made between each explanatory variable and the outcome variable, and the fulfillment of assumptions of chi-square was ascertained. Bivariate logistic regression analysis was conducted for the variables that fulfill the assumptions for chi-square, and all explanatory variables that have an association with the outcome variable at a *p* value of less than 0.25 were selected as candidates for multivariable analysis. Multicollinearity between the candidate variables was checked using the variance inflation factor (VIF ≥ 10). Finally, multivariable analysis was conducted to control for possible confounding variables. Model fitness was checked using the Hosmer and Lemeshow goodness-of-fitness test. The level of statistical significance was declared at a *p* value of <0.05. The odds ratio with its 95% CI was used to measure the degree of association between independent variables and the outcome variable.

#### 2.4.1. Data Quality Management

The questionnaire used in this study was initially prepared in English. It was then translated to Amharic, the common language in urban South Ethiopia, and to realize its validity, back translation to the English language was made by an expert having skills in both languages. Thorough training was provided to the data collectors and supervisors in advance. The questionnaire was pretested on a kebele that is not included in the sampled cluster in the study area on the 33 respondents (5% of sample size). Based on the pretest, questions were revised and edited, and those found to be unclear or confusing were modified. Every day, 10% of the completed questionnaires were reviewed and checked for completeness and relevance by the supervisors and a principal investigator and the necessary feedback was offered to data collectors the next morning before the actual procedure. Each woman was interviewed in a separate private place to avoid social desirability bias and protect the victims from further violence.

### 2.5. Measurement of Variables

The outcome variable of this particular study is an attitude that justifies wife-beating. Women were asked whether they justify if their husbands beat them in five conditions (goes out without telling him, neglects the children, argues with him, refuses to have sex with him, and burns the food) as “Yes” or “No” questions. Those who responded affirmatively to at least one of the questions (“Yes”) were considered as having an attitude that justifies wife-beating (favorable attitude) or not (unfavorable attitude) otherwise. In the SPSS, this was coded as “1” for those having a favorable attitude towards wife-beating and “0” for those having an unfavorable attitude.

The independent variables and other descriptive variables measured in the study which demand further clarity were presented as follows:

1. In this study, an intimate partner refers to a current spouse (husband).
2. Currently married women refer to those women who have been married and are not either divorced, widowed, or separated during data collection.
3. Intimate partner violence among currently married women (15–49) was measured as it exists when women reported that they experienced either of physical, psychological, or sexual forms of violence; otherwise, women were regarded as not having experienced an IPV [19].
4. Women were regarded as having experienced physical, sexual, and psychological violence if they had positively responded to either of the physical, sexual, and psychological violence acts presented to them in the questionnaire, respectively; otherwise, they were regarded as having not experienced any physical, sexual, and psychological violence, respectively [19].
5. Current intimate partner violence: when either of the physical, sexual, or psychological violence acts was experienced 12 months preceding the survey, such women were considered as having current intimate partner violence, respectively, whereas when it happened at any time before the preceding 12 months in their marital life, they were regarded as having lifetime violence [19].
6. Food insecurity status was measured using the recommendations of food insecurity measurement in the Food Insecurity Experience Scale. According to the scale, women respond on their perspectives about the last 12 months food conditions at their household level on six items assessing whether household members worried about food procurement, compromised on quality or variety, reduced quantity, skipped meal, and experienced hunger or not. When women reported as they or their families had not experienced any of the food insecurity conditions, their household was regarded as food secure; otherwise, it was regarded as a food-insecure household [21].
7. Household-level decision making was measured from a question having 4 decision issues at household (use of money and healthcare for self, major household purchases, and visits to family) with alternatives in scores from 0 to 2 where zero refers to a decision made to the woman by any other body, 1 refers to a joint decision with a partner or any other person, and 2 refers to a sole decision made by the woman. The total score of decision-making index was made in 0 to 8 limits. Women who scored 4 and above were regarded as having high decision-making power, and those who scored less than 4 were regarded as having a low decision-making power [19].
8. Partner's (husband's) drug use was measured as a user when a woman reported that her husband or partner was either a user of alcohol or khat; otherwise, it was regarded as not using a drug [19].

## 3. Ethics Approval and Consent to Participate

Ethical clearance was obtained from the Ethical Review Committee of Arba Minch College of Health Sciences. Letter of permission was obtained from the town's health office and each selected kebeles. Written consent on willingness to participate in the study was obtained from the study subjects. The questionnaire was anonymous, and there was not any other identifier included on the questionnaire.

## 4. Results

### 4.1. Individual and Household-Level Characteristics of the Study Participants

Out of the estimated samples of 737, 696 currently married women participated in the study yielding a response rate of 94.4%. The mean age of the participants and their partners was 31 ± 7 and 37 ± 11, respectively. About 474 (68.1%) of them had secondary and above education, and 494 (71.0%) had a family size of 4–7. About two-thirds (62.2%) and (61.1%) of them were under 30 years and from food-insecure households, respectively (Table 1).

### 4.2. Attitude of Women towards Wife-Beating

Overall, 59.5% (95% CI: 55.6–62.9%) of women in the study justified that wife-beating in on the five occasions was appropriate. Of the 429 women from food-insecure households, 297 (42.6%) had justified wife-beating on the five occasions as appropriate (favorable attitude) and the rest 132 (19.0%) had an unfavorable attitude towards wife-beating on the five occasions. However, 117 (16.8%) and 150 (21.6%) of the women had favorable and unfavorable attitudes among those from food-secure households, respectively. A significant difference was reported among women from food-secure and -insecure households (*x*^2^*p* value < 0.001).

Concerning the other characteristics of women, 239 (34.3%) and 109 (15.7%) of them had favorable and unfavorable attitudes, respectively, towards wife-beating among those who experienced intimate partner violence in the last 12 months (*x*^2^ test *p* value < 0.001). Of the 431 (61.5%) who experienced intimate partner violence ever, 295 (42.4%) and 136 (19.5%) of them were having favorable and unfavorable attitudes towards wife-beating, respectively (*x*^2^ test *p* value < 0.001). Of 190 (27.4%) who experience all forms of violence, 137 (19.8%) and 53 (7.6%) of them had favorable and unfavorable attitudes towards wife-beating, respectively (*x*^2^ test *p* value < 0.001) (Table 2).

### 4.3. Women's Attitude towards Wife-Beating

In the binary logistic analysis marriage planner, age difference among couples, household food security status, women's occupation, family size, partner's educational status, women's age, partner's age, household wealth status, and household decision making were the explanatory variables selected as candidates for multivariable logistic regression analysis. In the final model of multivariable logistic regression analysis food security status, marriage arranger/planner, age difference, family size, women's age, and household wealth index were significantly associated with women's attitude toward wife-beating.

Women from food-insecure households had 3.22 times higher odds of having a favorable attitude towards wife-beating compared with those from food-secure households (AOR = 3.22 (2.27, 4.56)). Women whose marriage was arranged by others (family members or any significant others) had 1.93 times higher odds of having a favorable attitude towards wife-beating compared with those whose marriage was arranged by both partners (AOR = 1.93 (1.11, 3.36)). The age difference between couples is also significantly associated with women's attitude towards wife-beating. Those who have 10 or more years' difference had 0.58 times lower odds having a favorable attitude towards wife-beating when compared with those who have less than 10 years' difference with their partner (AOR = 0.58 (0.39, 0.85)). In this study, an increase in family size resulted in a significant increase in having a favorable attitude towards wife-beating. Women from households with a family size of 5 and above had 1.76 times higher odds of having a favorable attitude towards wife-beating when compared with a family size of less than five (AOR = 1.76 (1.13, 2.77)). Age was the other factor that showed a significant association with women's attitude towards wife-beating. Younger women under 30 and those in the age ranged between 30 and 39 years had 1.66 and 2.34 times higher odds of having a positive attitude towards wife-beating compared with those 40 years and above (AOR = 2.34 (1.31, 4.19)). Finally, households with a high wealth index had 0.43 times lower odds of having a positive attitude towards women beating compared with households with a low wealth index (AOR = 0.43 (0.25, 0.75)) (Table 3).

## 5. Discussion

This study tried to elucidate the proportion of women with a supportive attitude towards wife-beating and factors associated with their justification. The justification of wife-beating by more than half of women in the study indicates that women themselves have taken wife-beating as an acceptable life event. This is relatively lower than a previous report from Northern Ethiopia and a study from Uganda [7, 9]. The higher acceptance in the two studies could be related to the inclusion of rural women. This present study was based in an urban environment where women can likely be educated, employed, exposed to mass media and, thus, can be aware of their sexual and reproductive health rights, are closer to the legal system, and enjoy a relaxation of sociocultural restrictions compared to women living in rural areas. However, the finding is very high seen in light of an ideal expectation of no acceptance among women and from recent reports from Zimbabwe [4] and nationwide data from the Ethiopian Demographic and Health Survey [15]. Even if variations can exist for various reasons, the consistent high prevalence of acceptance towards partner's beating in these African countries implies the degree of submissiveness among women themselves towards the already existing patriarchal culture. Noteworthy, the high degree of acceptance of wife-beating in the study area, in particular, the existence of a wide gap from the urban average from the EDHS, needs further qualitative exploration.

In addition to it being a significant predictor to IPV in recent literature, both in the study area and elsewhere [19, 23–26], household food insecurity was found to be a significant factor to affect women's justification towards wife-beating. This is plausible in the spirit that women trying to fulfill their household food conditions might be averted from rationalizing about their rights. When food insecurity is a concern, women might have mental inability and may become tolerant to violence. Such women might have favorable attitude towards beating from their partner [12, 13]. Repressed behind this is the issue of economic dependency of women on their partners. In the developing world, many women who are unemployed and uneducated look the hands of their husbands to secure food for their families which fuels the dependency of women on men and might in turn lead them to the acceptance of violence acts perpetuated by their husbands. It could also be related with a culture which gives men autonomous in many basic life decisions including resources which lead women usually to keep hands off their partner. As a consequence, women are usually expected to execute decisions made by their partner.

Another independent factor which showed a significant association with women's attitude towards wife-beating in this study was family size. The higher the family size, the more tolerance women showed can also be explained in a related fashion to the household food insecurity situation. When women have more children, they always get worried only about their children. They start to live only for the care and welfare of their child by tolerating any violence occurring to them and develop tolerance to the violence acts. They consider it as a “sacrifice to their children” as the culture itself gives child rearing and bearing as a major role of women. Even if the mother is expected to be physically closer to her child, the husband is expected to be the bread winner. Women in many cultures are incapable of caring, feeding, and educating their child independently since access to and distribution of assets is not treated equally to both genders. Needless to say, this gets worse when the number of children is higher, forcing women to tolerate the existing violence. A consistent finding was also reported from a previous study in Ethiopia [15]. Higher odds of acceptance to wife-beating among women below 40 years of age than those above 40 years of age was consistent with previous studies from Ethiopia, sub-Saharan African countries, Zimbabwe, and Bangladesh [7, 9, 11, 15]. As women get older, they might have a greater exposure to make social networks with neighbors and the society at large and, thus, would develop the tendency to refuse wife-beating. This reveals that gaining self-confidence and protecting own interest is the function of getting familiar with and establishing social networking within the community. On the other hand, it might also be related to their previous bad experiences from long time of exposure with their partners. The different experiences they acquire lead to having a lower favorable attitude towards wife-beating. The other possible explanation might also be as age increases, a better understanding of the legal system could also be there.

Likewise, in the present study, household wealth also showed a significant association with women's attitude towards wife-beating. Women from households with a lower wealth tertiles had a favorable attitude towards wife-beating compared with those in the higher tertiles. The finding is in line with findings from previous studies from sub-Saharan Africa, Nigeria, and Zimbabwe [7, 9, 14]. This might be explained as economic development creates opportunities for decision making through accessing information through many channels. Women tend to be disadvantaged compared to men in terms of their access to and control of the means of production and their welfare, particularly in low-income families. Such women are more likely to be undernourished, undereducated, overworked, and underpaid than their male partners and might be more tolerant to violence in the specified conditions.

A higher odds of having had a favorable attitude was reported among women whose marriage was arranged by another person and not by the will of the couples. In the study published earlier from this project, these groups of women faced higher odds of violence in the study area [19]. Women who accepted a marriage arranged by a third person would likely be those who share the societal attitudes in an “arranged marriage” and, hence, might have a favorable attitude towards wife-beating. In most cases, marriage is arranged by families in Ethiopia. Marriage is proposed through negotiation between elders from the prospective groom's family and the father of the prospective bride. When it is acceptable, families from both sides negotiate marital exchanges and set a wedding date [27].

## 6. Conclusions

A high magnitude of receptive attitude towards wife-beating was reported in the present study in an urban context of Southern Ethiopia. Various modifiable independent factors showed a significant association with the receptive attitude among women of the study. One of the important findings is the association between household food conditions and women's attitude towards wife-beating. Interventions targeting the improvement of women's attitude towards wife-beating should target the traditional norms of arranged marriage, women's household food conditions, and family size.

## Figures and Tables

**Table 1:** Individual and household characteristics of currently married women at Arba Minch town, Southern Ethiopia, March 2017.

Variables	Category	Frequency	Percentage
Marriage arranged by	Both partners	611	87.8
	Others^*∗*^	85	12.2

Age difference with the partner	<10 yrs	516	74.1
	10 or more yrs	180	25.9

Household food security	Food secure	267	38.4
	Food insecure	429	61.6

Women occupation	Housewife	308	44.4
	Government employee	203	29.3
	Student, daily laborer	63	9.1
	Merchant, NGO employee	120	17.3

Partner's occupation	Government employee	310	44.7
	Student, daily laborer	78	11.2
	Merchant, NGO employee	259	37.3
	Others	47	6.8

Family size	0–3	146	21.0
	4–7	494	71.0
	8 and above	54	7.8

Women's educational status	No formal education	128	18.4
	Primary-level education	94	13.5
	Secondary education and above	474	68.1

Partner's educational status	No formal education	126	18.1
	Primary-level education	401	57.6
	Secondary education and above	169	24.3

Woman's age	Less than 30	433	62.2
	30–39	171	24.6
	40 and above	92	13.2

Partner's age	Less than 30	248	35.6
	30–39	182	26.1
	40 and above	266	38.2

Household wealth index	Low	248	35.6
	Middle	359	51.6
	High	89	12.8

Household decision making	Low decision making	126	18.1
	High decision making	570	81.9

Partner drinks alcohol	Yes	264	37.9
	No	432	62.1

Partner chews khat	Yes	160	23.0
	No	536	77.0

Partner's history of drug use	Not a user	383	55.0
	User	313	45.0

Exposure to mass media	Yes	626	89.9
	No	70	10.1

Years in marriage	<10 years	423	60.8
	≥10 years	253	36.4
	Do not know	20	2.9

^*∗*^Parents, relatives, siblings, community elders, and religious leaders.

**Table 2:** Comparison of women's attitude towards wife-beating by their characteristics at Arba Minch town, Southern Ethiopia, 2018.

S. no.	Variable	Category	Attitude on wife-beating		*p* value
			Favorable Num (%)	Unfavorable Num (%)	
1	Food security status	Food secure	117 (16.8)	150 (21.6)	0.001
		Food insecure	297 (42.6)	132 (19.0)	

2	Intimate partner violence in the last 12 months	No	175 (25.1)	173 (24.9)	0.001
		Yes	239 (34.3)	109 (15.7)	

3	Intimate partner violence ever	No	119 (17.1)	146 (21.0)	0.001
		Yes	295 (42.4)	136 (19.5)	

4	All three forms of violence ever	No	276 (39.8)	227 (32.8)	0.001
		Yes	137 (19.8)	53 (7.6)	

5	Physical violence ever	No	166 (24.0)	190 (27.4)	0.001
		Yes	247 (35.6)	90 (13.0)	

6	Psychological violence ever	No	149 (21.5)	169 (24.4)	0.001
		Yes	264 (38.1)	111 (16.0)	

7	Sexual violence ever	No	241 (34.8)	200 (28.9)	
		Yes	172 (24.8)	80 (11.5)	

8	Marriage arrangement	Both	351 (50.4)	260 (37.4)	0.001
		Other	63 (9.0)	22 (3.2)	

9	Family size	<5	315 (45.3)	237 (34)	0.001
		≥5	99 (14.2)	45 (6.5)	

10	Partner's education	No formal	34 (4.9)	27 (3.9)	0.001
		Primary	42 (6.0)	23 (3.3)	
		Secondary	253 (36.4)	148 (21.3)	
		Other	85 (12.1)	84 (12.1)	

11	Age of the partner	<30	154 (22.1)	94 (13.5)	0.001
		30–39	119 (17.1)	63 (9.1)	
		40 and above	141 (20.2)	125 (18.0)	

12	Wealth index	Low	181 (26)	67 (9.6)	0.001
		Middle	182 (26.1)	177 (25.4)	
		High	51 (7.4)	38 (5.5)	

13	Age difference	<10 yrs difference	322 (46.3)	194 (27.9)	0.001
		10 and more yrs. difference	92 (13.2)	88 (12.6)	

14	Women's occupation	Housewife	197 (28.3)	111 (16)	0.001
		Others	217 (31.2)	171 (24.5)	

**Table 3:** Factors associated with attitude towards wife-beating among currently married women at Arba Minch town, Southern Ethiopia, 2020.

S. no.	Variables	Category	Attitude towards wife-beating		COR, 95% CI	AOR, 95% CI
			Favorable Num (%)	Unfavorable Num (%)		
1	Marriage arranged by	Both	351 (50.4)	260 (37.4)	1	1
		Others^+^	63 (9)	22 (3.2)	2.12 (1.27, 3.54)^a^	1.93 (1.11, 3.36)^b^

2	Age difference	Less than 10 years	322 (46.3)	194 (27.9)	1	1
		10 or more years	92 (13.2)	88 (12.6)	0.63 (0.45 , 0.88)^a^	0.58 (0.39, 0.85)^b^

3	Household food security	Food secure	117 (16.8)	150 (21.6)	1	1
		Food insecure	297 (42.7)	132 (19.0)	2.88 (2.11, 3.96)^a^	3.22 (2.27, 4.56)^b^

4	Women occupation	House wife	197 (28.3)	111 (15.9)	1	1
		Others^++^	217 (31.2)	171 (24.6)	0.72 (0.53, 0.97)^a^	0.77 (0.54, 1.08)

5	Family size	<5	315 (45.3)	237 (34.1)	1	1
		5 and above	99 (6.5)	45 (14.2)	1.65 (1.12, 2.45)^a^	1.76 (1.13, 2.77)^b^

6	Partner's educational status	No formal education	76 (10.9)	50 (7.2)	1	1
		Primary-level education	253 (36.4)	148 (21.3)	1.13 (0.75, 1.69)	1.36 (0.85, 2.17)
		Secondary and above education (*p* value = 0.08)	85 (12.2)	84 (12.1)	0.66 (0.42, 1.06)	0.75 (0.44, 1.28)

7	Women's age	Less than 30	256 (36.8)	177 (25.4)	1.38 (0.88, 2.17)	1.66 (0.97, 2.82)
		30–39	111 (15.9)	60 (8.6)	1.77 (1.06, 2.96)^a^	2.34 (1.31, 4.19)^b^
		40 and above	47 (6.8)	45 (6.5)	1	1

8	Partner's age	≤30	154 (22.1)	94 (13.5)	1.45 (1.02, 2.06)^a^	1.36 (0.76, 2.42)
		31–39	119 (17.1)	63 (9.1)	1.67 (1.14, 2.47)^a^	1.63 (0.91, 2.94)
		40 and above	141 (20.3)	125 (18.0)	1	1

9	Household wealth index	Low	181 (26.0)	67 (9.6)	1	1
		Middle	182 (26.1)	177 (25.4)	0.38 (0.27, 0.54)	0.31 (0.21, 0.44)^b^
		High	51 (7.3)	38 (5.5)	0.49 (0.30, 0.83)	0.43 (0.25, 0.75)^b^

10	Household decision making	Low decision making	84 (12.1)	42 (6)	1	1
		High decision making	330 (47.4)	240 (34.5)	0.68 (0.46, 1.03)	0.75 (0.47, 1.18)

^+^Family members, any significant others. ^a^Significant at *p* value less than 0.25 in the bivariate analysis. ^b^Significant at *p* value less than 0.05 in the multivariable analysis after adjusting for food security status, age difference, decision making by women, women occupation, marriage arranger, family size, women's age, age of the partner, wealth index of households, and education status of the partner.

## Data Availability

The datasets used and/or analyzed during the current study are available from the corresponding author on reasonable request.
